# Microarray profiling of differentially expressed lncRNAs and mRNAs in lung adenocarcinomas and bioinformatics analysis

**DOI:** 10.1002/cam4.3369

**Published:** 2020-09-01

**Authors:** Fei He, Liping Huang, Qiuping Xu, Weimin Xiong, Shuang Liu, Huimin Yang, Wanting Lu, Rendong Xiao, Zhijian Hu, Lin Cai

**Affiliations:** ^1^ Department of Epidemiology and Health Statistics School of Public Health Fujian Medical University Fuzhou China; ^2^ Fujian Provincial Key Laboratory of Environment factors and Cancer Key Laboratory of Ministry of Education for Gastrointestinal Cancer Fujian Medical University Fuzhou China; ^3^ Department of Basic Medicine Zhangzhou Health Vocational College Zhangzhou China; ^4^ Department of Thoracic Surgery The first affiliated hospital of Fujian Medical University Fuzhou China

**Keywords:** lncRNAs, lung adenocarcinomas, mRNA, TCGA

## Abstract

Long noncoding RNAs (lncRNAs) dysregulation leads to malignant progression of lung cancer. Our study profiled differentially expressed lncRNA and mRNA in tumor and normal tissues of lung adenocarcinoma (LUAD). Further, analysis of the gene expression profiles of LUAD tissues (n = 533) and normal tissues (n = 59) from The Cancer Genome Atlas (TCGA). A total of 138 lncRNAs were differentially expressed between LUAD tissues and normal tissues (false discovery rate [FDR] *q* < 0.05, fold change (FC) ≥ 2), a number of which are key regulators of multiple cancer and biological processes in humans. For example, lncRNA *A2M‐AS1* displayed the highest correlation with the co‐expressed mRNAs, indicating that it might play a key role in regulating differential gene expression in LUAD. The data from the current study of the comprehensive lncRNA expression profile in LUAD tissues provided useful information to guide the identification of potential LUAD biomarkers.

## Graphical abstract

We performed a study in 8 lung adenocarcinomas patients and validated our observations using The Cancer Genome Atlas (TCGA) dataset. We found that 138 lncRNAs were upregulated or down regulated similarly to those in our microarray study. The lncRNA–mRNA co‐expression network was constructed based on the correlation analysis. Our results revealed that the lncRNA A2M‐AS1 exhibited the highest correlation with the co‐expressed mRNAs, indicating that it might play a key role in regulating differential gene expression in lung adenocarcinomas patients.

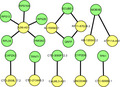

## 1 INTRODUCTION

Lung cancer is the deadliest cancer worldwide, accounting for 1.59 million cancer‐related deaths ^1^ and 13% of all newly diagnosed human cancers in 2012.^2^ In China, more than 529 000 cases died from the lung cancer in 2011, accounting for 25% of all deaths due to cancer, despite recent advancements in early detection, therapeutic options, and prevention.^3^ Based on histological and clinical criteria, lung cancer can be divided into small cell lung cancer (SCLC) and non‐small cell lung cancer (NSCLC), and lung adenocarcinoma (LUAD) is the most common histologic subtype of NSCLC. In recent years, the incidence of LUAD has increased and prognosis is usually poor due to the diagnosis commonly occurring at advanced stages of the disease. Thus, the evaluation and identification early detection biomarkers is of urgent need for predicting prognosis and improvement LUAD treatment responses.^4^, ^5^

Long noncoding RNAs (lncRNAs) are a class of molecules that regulate gene expression and protein synthesis, but their functions remains to be elucidated. Previous studies have suggested that aberrant expression and certain functions of lncRNAs are associated with the malignant progression of lung cancer.^6^, ^7^ For example, lung cancer‐associated lncRNA 6 (*LCAL6*) has been shown to be significantly upregulated in LUAD and could predict the survival of patients, while silencing of *LCAL6* inhibited cell survival in vivo and in vitro.^8^ Moreover, knockdown of the *LINC00511* and lncRNA *AFAP1‐AS1* reduced the malignant phenotypes of LUAD cells.^9^ Studies had shown that lncRNAs were closely related to LUAD, including *HOTAIR, MALAT1, GAS5, Sox2ot, BRAF, FLJ30679, LINC00511, CTC‐429P9.1, LINC01127, MIF‐AS1, RP11‐278J6.4,* and *RP11‐25K19.1*.^10^, ^11^, ^12^, ^13^, ^14^

Here, we have profiled the differentially expressed lncRNAs in eight paired LUAD tissues and corresponding normal tissues using a microarray, and compared our data against those in TCGA by using bioinformatic analyses. We aimed to provide a novel information regarding the use of lncRNAs as diagnostic and prognosis markers for LUAD.

## 2 MATERIALS AND METHODS

### 2.1 Human LUAD specimen collection and total RNA extraction

All experiments were approved by the Ethics Committee of Fujian Medical University (Fujian, China) ([2014] Fu Yi Ethics Review (No.98)) and all LUAD patients read and signed the consent form to participate in this study. The paired LUAD and corresponding normal tissues (approximately 5 cm away from the tumor lesion) were collected from eight eligible patients who lived in Fujian province for more than 10 years and underwent surgical resection of the tumor lesions at the first hospital, Fujian Medical University between June 2014 and March 2015. All patients were pathologically diagnosed with primary LUAD (ICD 33‐34).

Total RNA was isolated from these tissues with TRIzol reagents (Invitrogen), quantified using the NanoDrop ND‐8000 spectrophotometer (NanoDrop Technologies), and standard denaturing agarose gel electrophoresis, according to the manufacturer's instructions. Each RNA sample (total amount > 8 μg) was used for lncRNA and mRNA profiling analysis.

### 2.2 Microarray data production

In this study, we utilized the mRNA plus lncRNA Human Gene Expression Microarray V4.0 from CaptialBio Corp., which contains 40 914 human lncRNAs probes, 34,235 mRNA probes, and 68 control probes. Our RNA samples were subjected to synthesis of complementary DNA probes and hybridization to the microarrays. The microarray data were scanned and processed using Agilent Feature Extraction software (v10.7, Agilent Technologies, Inc) and the quality control, normalization, and summary was performed using the GeneSpring GX software (v11.5, Agilent Technologies, Inc). Gene profiling were submitted to the Gene Expression Omnibus repository with the accession number GSE130779. The data in GSE130779 were raw gProcessedSignal values and in current study, the gProcessedSignal data were normalized by 75% percentile of all probe values, and then, log2 transformed for further analysis.

### 2.3 Microarray analysis

The lncRNAs and mRNAs profiling were assessed using the Benjamini‐Hochberg procedure. Significantly data were defined as a False Discovery Rate (FDR) *q*‐value less than 0.05 and a twofold difference between the LUAD and corresponding normal lung tissues. Heatmaps, volcano plots, and scatterplots were created using TreeView software (Stanford University, Palo Alto, CA, USA). The pathway analysis and Gene Ontology (GO) by using DAVID (Beta; https://david‐d.ncifcrf.gov/) and the functions of these differentially expressed mRNAs and lncRNAs in LUAD tissues by using Reactome Database (http://www.reactome.org). Finally, the pairs of lncRNAs and mRNAs networks were constructed using Pearson correlation coefficients and visualized using Cytoscape software (http://www.cytoscape.org/).

### 2.4 Validation using TCGA

We downloaded RNA sequencing data of 533 tissues of LUAD and 59 nonmatching normal tissues from TCGA, with the exclusion criteria of incomplete data analysis. Differentially expressed lncRNAs were identified with edge R using an adjusted *P* value < .05 as the threshold. The data retrieved from TCGA were intersected with our microarray results and we then computed those intersected lncRNAs co‐expression with mRNAs using weighted gene co‐expression network analysis (WGCNA).^15^

Next, the enrichment of GO terms, Kyoto Encyclopedia of Genes and Genomes (KEGG) pathways by using DAVID. The functions of mRNAs and lncRNAs in LUAD were assessed by using Reactome Database. A *P* value < .05 was considered to be statistically significant. Then, we constructed the co‐expression network using Cytoscape 3.7.0., according to the differentially expressed mRNAs and lncRNAs.

### 2.5 Cell culture

Human lung cancer cell lines A549 (Catalog number: TCHu150), NCI‐H1975 (Catalog number: TCHu193), and PC‐9 (Catalog number: CBP60078) were obtainded from Cell Bank of the Chinese Academy of Sciences (Cobioer, Shanghai, China). The normal human lung cell line 16HBE (Catalog number: CBP60550, Cobioer, Shanghai, China) were obtained. Cells were cultured in RPMI‐1640 (Gibco, Carlsbad, CA, USA, Catalog number: C11875500BT) containing 10% of fetal bovine serum (Gibco, Carlsbad, CA, USA. Catalog number: 10099141C), 1% of penicillin and streptomycin (Hyclone, Logan, UT, USA. Catalog number: SV30010). Cells were grown at 37℃ in a humidified atmosphere of 5% of CO_2_.

### 2.6 Q‐PCR analysis

A total of 2 × 10^5^ cells per well were seeded in six‐well plates. After incubation for 48 hours at 37℃, RNAiso Plus reagent (Takara, Dalian, Liaoning, China. Catalog number: 9109) was used to extract total RNA. Total RNA was reverse transcribed using PrimeScript RT reagent kit (Takara, Dalian, Liaoning, China. Catalog number: RR037A) according to manufacturer's instructions. Quantitative PCR (Q‐PCR) was done using ABI 7500 Fast Real‐Time PCR System (Applied Biosystems, Carlsbad, CA, USA). The amount of experimental repetitions number of biological replicates per experiment were all three and each biological replicate had two technical replicates. Cycling conditions were 95°C for 3 minutes, 40 cycles of 95°C for 15 seconds, 60°C for 30 seconds. The Relative mRNA levels were determined using the 2^‐ΔΔCT^ method. The sequences of the primers are described in Table 1.

**TABLE 1:** The specific primer sequences for Q‐PCR are performed as follows

Gene	Primers (5′‐3′)
LINC00641	F: TAGTTTATTTGGCGTTTGGCTC R: TCCACCCCATTACATACCCATC
H19	F: GCGGGTCTGTTTCTTTACTTCC R: CTGCTGTTCCGATGGTGTCTT
CTD‐2517M22.14	F:TGTCACAGGCATTGATGTTGGC R:AGTTCCTTGGGAGTGGGGTC
RP11‐498J9.2	F:GAATCCTGACTGCCTGGACATG R:GTGACACTTCTTCAACATGGGG
GAPDH	F: ATGGGGAAGGTGAAGGTCG R: GGGGTCATTGATGGCAACAATA

Note

F, Forward；R, Reverse.

### 2.7 Statistical analysis

All data were represented as mean ± standard deviation (SD). Statistical analyses were conducted using the SPSS 25.0 and R 3.5.0. For data of qPCR, ln‐transformation has been done beforehand to approximate normal distribution, and then, the expression of these lncRNAs were presented as the mean ± SD. The analysis of variance (ANOVA) was used for comparisons of four independent groups and using LSD methods for post hoc multiple comparison tests. *P* < .05 indicated statistical significant.

## 3 RESULTS

### 3.1 Baseline demographic and clinical characteristics of LUAD patients

In this study, we obtained samples from eight LUAD patients, and their demographic and clinical characteristics are shown in Table 2. In brief, there were four females and four males with an average age of 54 years. Only two patients ever smoked tobacco. The average tumor size was 3.9 cm in diameter and there were five Stage I, one Stage II, and two Stage III LUAD.

**TABLE 2:** Baseline demographic and clinical characteristics of the study subjects

Subject ID	1	2	3	4	5	6	7	8
Age	56	41	60	60	56	44	58	59
Gender	Female	Female	Male	Male	Female	Female	Male	Male
Tumor size (cm)	4	2	6.7	4.5	4	4	3	3
Tobacco smoking	No	No	Yes	No	No	No	Yes	No
TNM stage	III	I	I	I	III	II	I	I

Abbreviation: TNM, tumor, lymph node, metastasis.

### 3.2 Differentially expressed lncRNAs and mRNAs in LUAD

**FIGURE 1:**
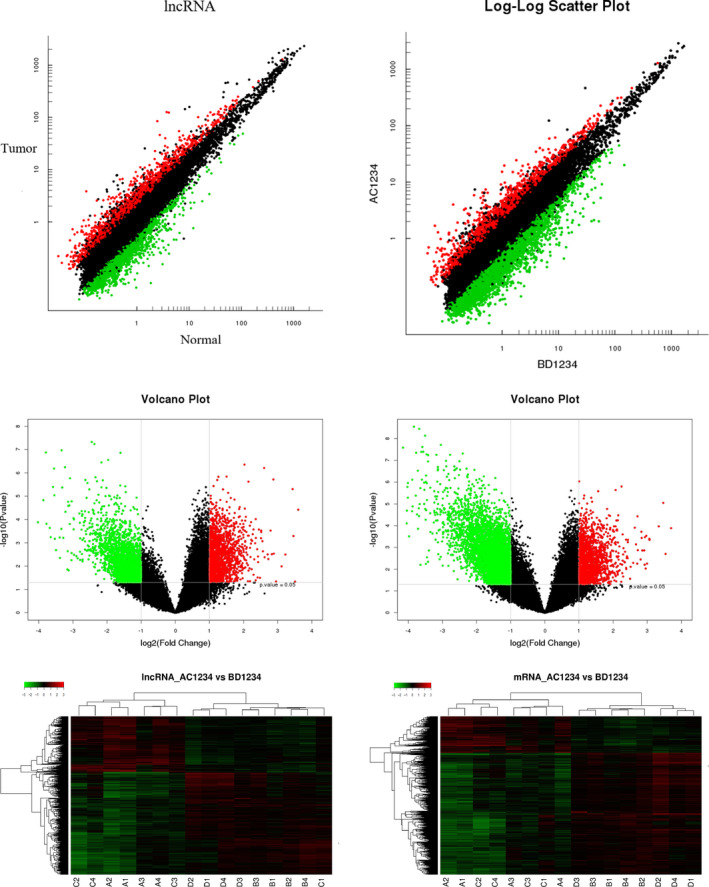
Identification of differentially expressed lncRNAs and mRNAs in LUAD tissues compared to corresponding normal lung tissues. The variation in lncRNA (A) and mRNA (B) expression between the two groups were assessed using Scatter plots. Differentially expressed lncRNAs (C) and mRNAs (D) were identified through Volcano Plot filtering. The red and green points in the plot indicate 2.0‐fold up‐ and downregulation of expression between the two groups, respectively. Heatmap and hierarchical clustering analysis were performed to examine distinguishable lncRNA (E) and mRNA (F) expression patterns. In the dendrogram, the red, green, black, and gray represent a high relative expression, a low relative expression, tumor tissue samples, and normal tissue samples, respectively

A total of 4,192 differentially expressed lncRNAs and 6,544 differentially expressed mRNAs (FDR *q* < 0.05, fold change ≥ 2) in the eight LUAD tissues. Specifically, compared with the corresponding normal lung tissues, there were 1,476 upregulated lncRNAs, 1,508 upregulated mRNAs, 2,716 downregulated lncRNAs, and 5,036 downregulated mRNAs in the tumor tissues. As shown in Figure 1A‐D, we summarized the lncRNAs and mRNAs with the greatest differential expression. Next, these differentially expressed lncRNAs and mRNAs were performed by using hierarchical clustering analysis, and heatmaps and dendrograms are shown in Figure 1E,F.

### 3.3 Classification of differentially expressed lncRNAs

Further, we classified these 2716 downregulated and 1476 upregulated lncRNAs according to their genetic orientation and location (sense, antisense, divergent, intergenic, and intronic). The lncRNAs could have different genome localizations and context, or various effects on DNA, as well as unique functional and targeting mechanisms. These data are shown in Table 3.

**TABLE 3:** Classification of the differentially expressed lncRNAs between the lung adenocarcinoma and normal tissues

lncRNA classification	Upregulated	Downregulated	Total
Sense lncRNA	329	567	896
Antisense lncRNA	312	539	851
Divergent lncRNA	108	160	268
Intergenic lncRNA	628	1037	1665
Intronic lncRNA	99	413	512

### 3.4 Establishment of the lncRNA‐mRNA co‐expression network

**FIGURE 2:**
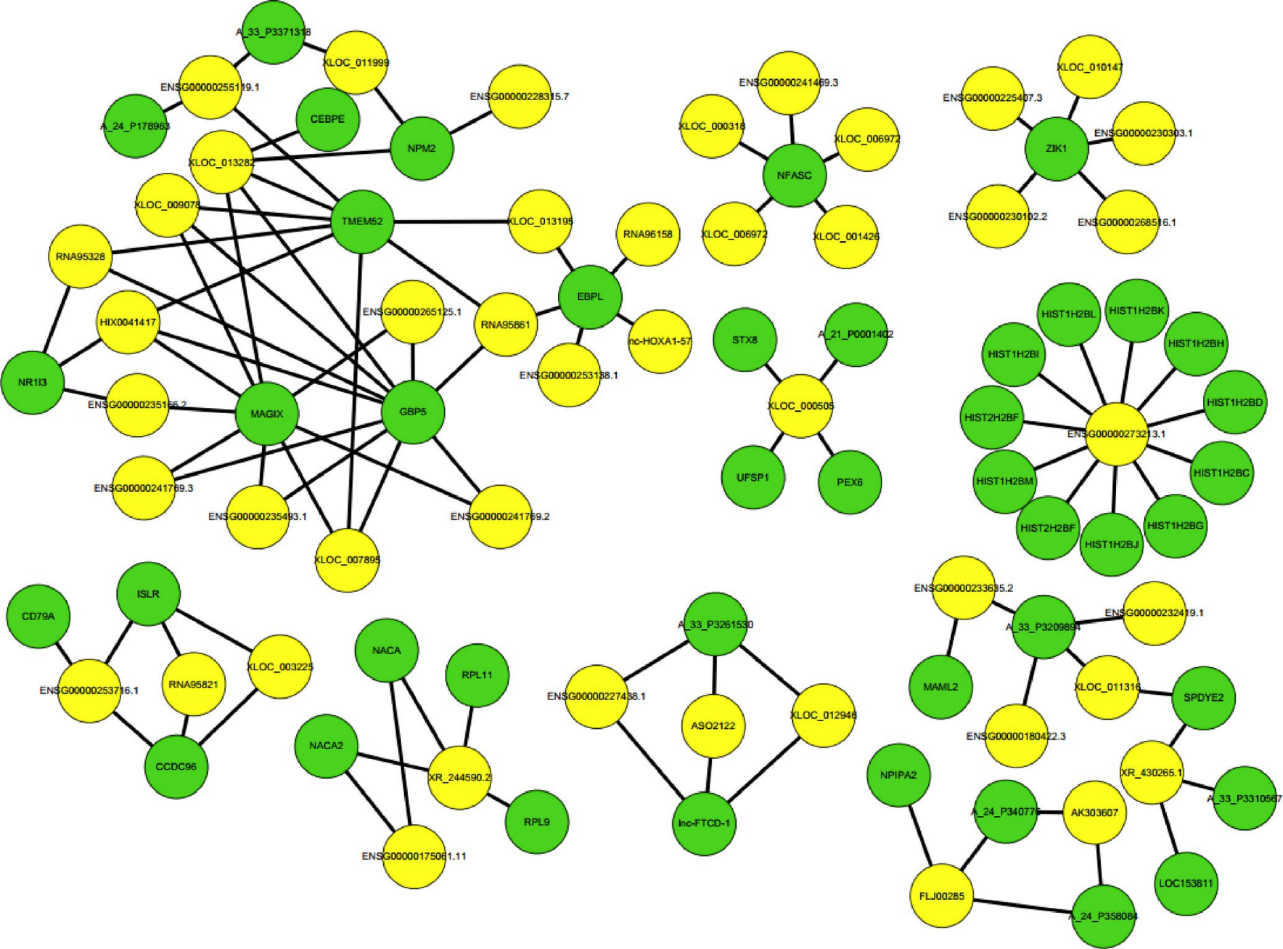
The lncRNA‐mRNA co‐expression network of the selected correlations. The yellow circles represent lncRNAs, while the green circles represent mRNAs. The node degree is denoted by size of the circle, while the edge indicates a co‐expression between an lncRNA and an mRNA in the context

Since lncRNA functions to regulate protein expression from mRNA, we established the network of lncRNA‐mRNA co‐expression according to Pearson's correlation analysis. We only included gene pairs that had an absolute Pearson's correlation coefficient value greater than 0.99. We found that 236 lncRNA‐mRNA pairs, and from these constructed an lncRNA‐mRNA co‐expression network (Figure 2). The network showed that one mRNA could correlate with several vice versa or lncRNAs, and there were two of particular interest: the lncRNA with the greatest number of mRNA neighbors was *ENSG00000273213.1*, which was associated with the expression of *HIST1H2* family members; and *TMEM52*, *GBP5*, and *MAGIX* were mRNAs with the most lncRNA neighbors in the network.

### 3.5 Gene ontology (GO) and pathway analyses

**FIGURE 3:**
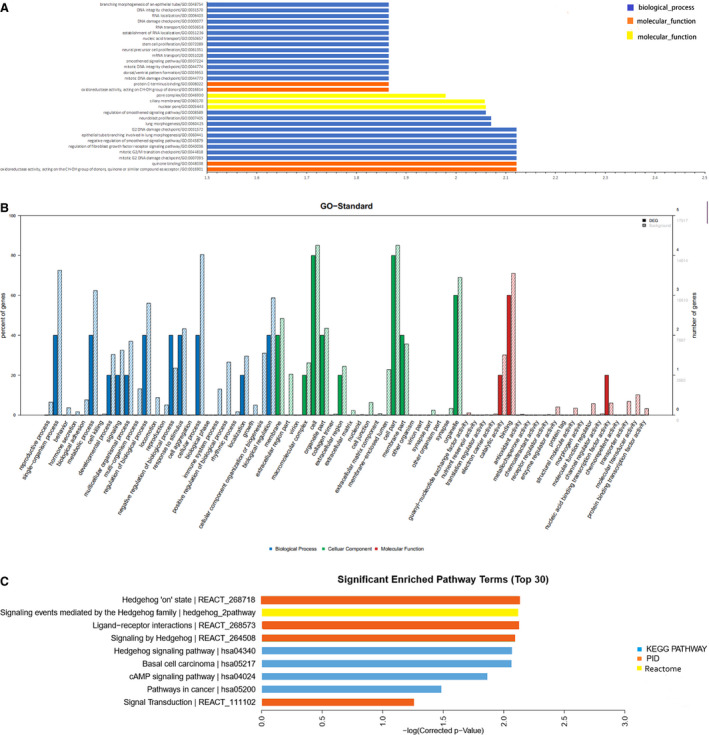
The GO and KEGG pathway analyses in LUAD. A, The top 30 GO terms enriched among the up‐ and downregulated mRNAs in lung adenocarcinoma (LUAD). B, The GO analysis of differentially expressed mRNAs with a twofold change in expression level in LUAD tissues. C, The most enriched pathways among the mRNAs in LUAD tissues

We conducted GO analysis of the 6,544 differentially expressed mRNAs to assess their functions and found that the significant GO terms were mainly associated with oxidoreductase activity, action on CH‐OH group of donors (GO:0016614), mitotic DNA damage checkpoint (GO:0044773), pore complex (GO:0046930), and dorsal/ventral pattern formation (GO:0009953) (Figure 3A). Moreover, our data showed that the G2 DNA damage checkpoint (GO:0007095), G2/M transition checkpoint (GO: 0044818), nuclear pore (GO:0005643), and ciliary membrane (GO:0060170) were the most enriched GO terms (Figure 3B). Furthermore, our result revealed that the signal transduction (REACT_111102), the cancer‐related signaling pathway (hsa05200), and the cAMP signaling pathway (hsa04024) were the most significantly regulated pathways (Figure 3C).

### 3.6 Comparison of our microarray data with those of the TCGA database

**FIGURE 4:**
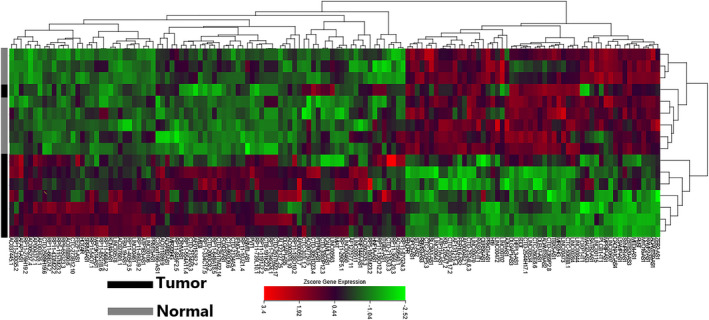
The 138 dysregulated lncRNAs in our microarray and TCGA data set data. The distinguishable lncRNA expression patterns that were further validated in the TCGA data sets by the hierarchical clustering and heatmap analyses. In the dendrogram, the red, green, black, and gray represent a high relative expression, a low relative expression, tumor tissue samples, and normal tissue samples, respectively

We compared our microarray profiling data with RNA sequencing data from TCGA and found that many of the lncRNAs that were up‐ or downregulated in our study were consistent with those that were differentially expressed between LUAD tissues and not‐matched normal tissues in TCGA. Compared to normal tissues, 138 dysregulated lncRNAs in the LUAD tissues, including 84 were upregulated and 54 were downregulated (*P* < .05, ≥2‐fold change) (Figure 4, Table 4). Surprisingly, the heterogeneity appear in diverse transcriptomics expression profile, so with respect to these 138 lncRNAs, sample C1 clusters with the normal tissue samples rather than the LUAD samples.

**TABLE 4:** The 20 dysregulated lncRNAs in our microarray and TCGA data sets

lncRNA	*FDR‐corrected P*	*P*	Fold change	Regulation
CTD‐2517M22.14	0.011193	0.000426	4.819499	Up
RP11‐10J21.4	0.017169	0.001072	4.645266	Up
RP11‐498J9.2	0.006585	0.000113	4.13408	Up
ASMTL‐AS1	0.015743	0.000911	4.132243	Up
AFAP1‐AS1	0.009646	0.000303	4.095078	Up
MIAT	0.016612	0.001008	3.470605	Up
CTB‐191K22.6	0.054829	0.009596	3.357024	Up
AC005237.4	0.008428	0.000221	3.345144	Up
RP11‐290F5.1	0.072095	0.015096	3.317507	Up
RP11‐794G24.1	0.088811	0.021267	3.298012	Up
LINC00641	0.011284	0.000441	5.642809	Down
CTC‐232P5.3	0.047801	0.007569	4.361985	Down
H19	0.045314	0.006849	4.201702	Down
ADAMTS9‐AS1	0.013292	0.000639	4.184906	Down
CTD‐2369P2.8	0.050708	0.008365	4.100527	Down
EGOT	0.037106	0.004814	4.064008	Down
RMST	0.040275	0.00561	3.930113	Down
LINC00284	0.026519	0.002595	3.755476	Down
CTD‐2544H17.1	0.091803	0.022417	3.722015	Down
LINC00607	0.024191	0.002171	3.635472	Down

**FIGURE 5:**
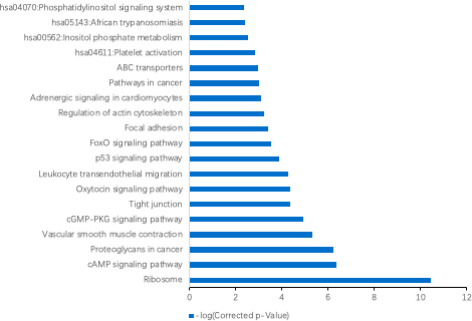
The KEGG pathway analysis of LUAD in the TCGA data sets. The corrected *P* values showed the significance of the correlation between the pathway and LUAD

We then performed GO and KEGG functional enrichment analysis for 628 mRNA co‐expressed with the 138 lncRNAs to reveal the potential functions. Our result showed that lncRNA‐mRNAs co‐expression network was significantly enriched in the following pathways: the ribosome (KEGG:03010), cAMP signaling (KEGG:04024), proteoglycans in cancer (KEGG:05205), vascular smooth muscle contraction (KEGG:04270), cGMP‐PKG signaling (KEGG:04022), and tight junctions (KEGG:04530) (Figure 5). These differentially expressed mRNAs were enriched in the following the GO terms: nuclear‐transcribed mRNA catabolic process, nonsense‐mediated decay (GO:0000184), the SRP‐dependent protein targeting to the membrane (GO:0006614), translational initiation (GO:0006413), muscle contraction (GO:0006936), viral transcription (GO:0019083), rRNA processing (GO:0006364), smooth muscle contraction (GO:0006939), and Rho protein signal transduction (GO:0007266).

### 3.7 The TCGA validated lncRNA‐mRNA co‐expression network

**FIGURE 6:**
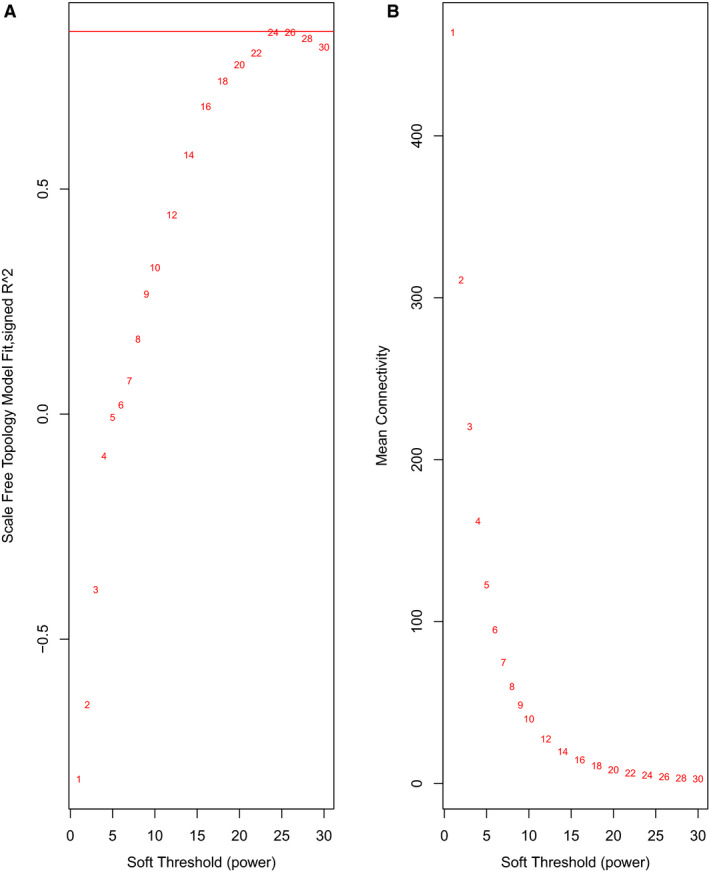
The correlation coefficients of the lncRNA‐mRNA co‐expression network by soft threshold filtering. A, The higher the correlation coefficients of log (k) and log (pk) indicates that the network has a more consistent scale‐free network distribution. B, A higher mean centrality implies that the lncRNA‐mRNA acts as a bridge to connect the different network components and control communication

**FIGURE 7:**
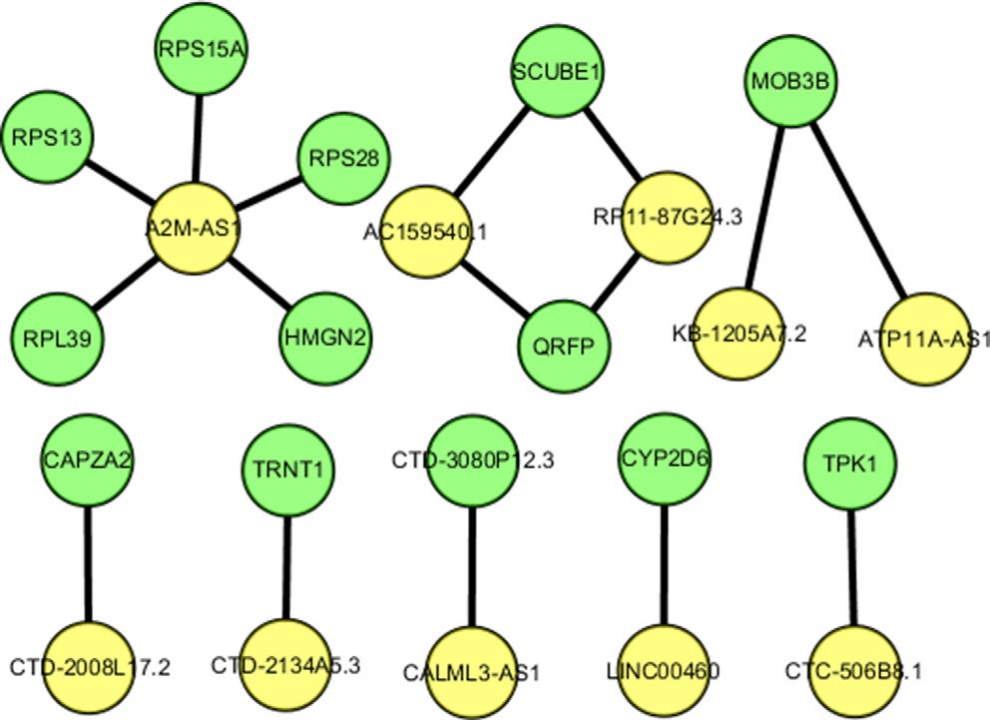
The differentially expressed mRNAs and lncRNAs co‐expression network in LUAD. This sample network contains 16 matched lncRNA‐mRNA pairs for 10 differentially expressed lncRNAs and 13 differentially expressed mRNAs. The red diamonds denote lncRNAs, while the green circles denote mRNAs. An edge represents a co‐expression relationship between an lncRNA and an mRNA in the network

We performed WGCNA analysis to identify gene co‐expression networks associated with LUAD progression. A higher correlation coefficient value of log (k) and log (p(k)) indicates that the network has a more consistent scale‐free network distribution (Figure 6A). The higher mean centrality data implicate that they could act as a bridge to connect the different network components and control the lncRNA‐mRNA regulation or protein communication (Figure 6B). For example, the β = 22 related co‐expression network consisted of 16 matched lncRNA‐mRNA pairs representing 10 differentially expressed lncRNAs and 13 mRNAs (Figure 7). In this network, the lncRNA *A2M‐AS1* exhibited the highest correlation with the co‐expressed mRNAs, suggesting that *A2M‐AS1* may play a critical role in the regulation of gene expression in LUAD.

### 3.8 Validation of results in human LUAD cell lines

**FIGURE 8:**
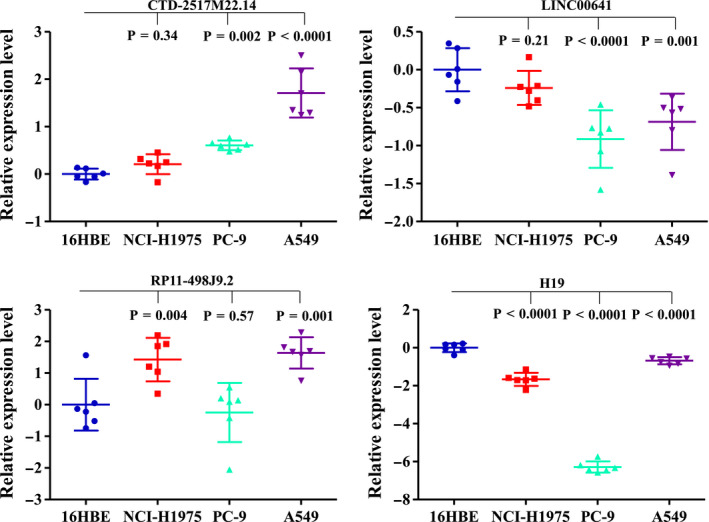
Expression of selected lncRNAs in LUAD cell lines. The expression level of lncRNAs in three LUAD cell lines and normal lung cell were detected by Q‐PCR. GAPDH was used as an internal control. The number of biological replicates per experiment was three and each biological replicate had two technical replicates

To further confirm the differential expression of lncRNAs in LUAD, the expression of *LINC00641*, *H19*, *CTD‐2517M22.14*, *RP11‐498J9.2* were examined using Q‐PCR in human LUAD cell lines A549, NCI‐H1975, and PC‐9, and normal human lung 16HBE cells. As shown in Figure 8A, both the expression of *CTD‐2517M22.14* and, *RP11‐498J9.2* were significantly increased in human LUAD cell lines compared with 16HBE cells (*P* < .0001), which confirmed their increase in LUAD tissues (Figure 4). In contrast, we also found that the expression of *LINC00641* and *H19* was significantly decreased in A549, NCI‐H1975, and PC‐9 cells compared with normal 16HBE cells (Figure 8, *P* < .0001), whose expression in LUAD was significantly decreased compared with 16HBE cells.

## 4 DISCUSSION

Gene expression profiling could help to identify the critical role of noncoding transcripts in the malignant progression of various human diseases, including LUAD. Alterations in lncRNA expression and function are associated with the development of several cancers, and they may act as oncogenes or tumor suppressors to regulate the expression of genes critical for cell growth and apoptosis through histone protein modification, chromosome reprograming, and RNA decay, miRNA sponging.^16^ Compared with protein‐coding genes, lncRNAs exhibit a notably higher degree of cell‐ and tissue‐specific expression patterns,^17^ suggesting specific regulatory roles and biological functions of lncRNAs within individual tissue types.

Clinically, compared with many other human cancers, LUAD has a higher incidence rate and poor prognosis, with a five‐year overall survival rate of approximately 20%.^18^ Despite the continuous refinement of the staging system used to guide treatment and predict the outcome of LUAD patients, molecular heterogeneity varies considerably in LUAD. Thus, molecular markers to potentially diagnose LUAD have been systematically studied clinically over the past few years, including some lncRNA profiling studies.^19^, ^20^, ^21^ However, information about specific lncRNAs associated with LUAD is still limited. In order to better assess the tissue‐specific regulatory network of lncRNAs in LUAD, we profiled lncRNAs and mRNAs in LUAD tissue samples to identify significant GO terms and to construct a functional lncRNA‐mRNA network. Indeed, our current microarray analysis identified many differentially expressed mRNAs and lncRNAs associated with various cell biological processes, including those encoding proteins involved in the malignant progression of cancers. Our co‐expression analysis a cluster of three mRNAs with the most lncRNA neighbors in the network: *TMEM52*, guanylate‐binding protein 5 (*GBP5*), and *MAGIX* *TMEM52* has been shown to be downregulated in pancreatic cancer,^4^, ^22^ while a recent study by Patil et al have demonstrated that the interferon‐inducible immunoregulatory gene *GBP5* is highly expressed in gastric adenocarcinoma, suggesting that it plays an important role in tumor pathobiology.^23^ The Human Protein Atlas indicates that *MAGIX* mRNA is moderately expressed in liver, gastric, pancreatic, and thyroid cancers. The co‐expression analysis also identified that the lncRNA *ENSG00000273213.1* has the greatest number of mRNA neighbors. This lncRNA, which was able to predict survival of colon adenocarcinoma patients in a previous study,^24^ was positively correlated with the expression of *HIST1H2* family members, indicating that it plays a role in epigenetic deregulation in LUAD.

Our GO term enrichment analysis indicated that the genes with significant differential expression between LUAD and corresponding normal lung tissues are involved in regulation of cell cycle progression, including G2 DNA damage checkpoint (GO:0007095) and G2/M transition (GO:0044818), while our gene pathway analysis showed that the cancer‐related pathways and the cAMP signaling pathway (has 04024) were the most significantly upregulated in LUAD. Furthermore, our current data were consistent with those of the TCGA data set. Among these lncRNAs, *AFAP1‐AS1*, *HOTAIR*, and *PVT1* have been reported to be upregulated in NSCLC and to promote cell invasion and proliferation.^25^, ^26^, ^27^ In addition, our KEGG analysis suggested that these altered lncRNA‐related protein‐coding genes could participate in multiple biological processes, such as the nuclear mRNA catabolic process, nonsense‐mediated decay, muscle contraction, and viral transcription, and so on. Several tumor‐related pathways were also identified in our current study, such as the tight junction/p53 signaling pathway and ribosome/cAMP signaling pathway in cancer. However, comparing the GO and pathway analysis results between the LUAD patient samples and the validated lncRNAs, we found that only a few biological processes and pathways were shared between these two different transcript resources. This discrepancy may be due to the different study populations or evaluation criteria of the clinical variables between the two studies, suggesting that further individual lncRNA validation is necessary.

To identify the functional significance of lncRNAs in LUAD, we also used WGCNA to construct an lncRNA‐mRNA co‐expression network with 138 TCGA‐validated lncRNAs with significant correlations. The network was composed of 10 lncRNAs and 13 mRNAs, indicating that one lncRNA could correlate with a large number of target mRNAs and vice versa. And the aberrant expression of lncRNAs might lead to an extensive variation in gene expression through lncRNA‐mRNA crosstalk interactions, further implicating the important role of lncRNAs in the malignant progression of LUAD. We identified that the lncRNA *A2M‐AS1* exhibited the highest correlation with the co‐expressed mRNAs, suggesting that *A2M‐AS1* dysregulation may play a key role in regulating differential gene expression in LUAD. Further in‐depth analysis of the lncRNA *A2M‐AS1* is necessary in the future.

We also selected two of the most strongly upregulated lncRNAs (*CTD‐2517M22.14* and *RP11‐498J9.2*) and downregulated lncRNAs (*LINC00641* and *H19*) for validation. The level of *LINC00641* and *H19* in lung cancer cell lines was found to be evidently lower than that in normal lung cell lines. Together with the increased expression of *CTD‐2517M22.14* and *RP11‐498J9.2* in lung cancer cell lines, we suggest that the present results could confirm lncRNAs signature may play a critical role in LUAD pathogenesis.

In conclusion, this study is the first to present both lncRNAs and mRNAs expression profiles from LUAD patients. These results suggested that abnormally expressed lncRNAs are key molecular of gene expression and have important biological effects. However, a limitation of the present study should be acknowledged. Since noncoding RNAs were several folds more abundant than those of protein‐coding genes, only lncRNAs were included in the validation study. Therefore, the current study only a limited part of the whole transcription alteration associated with LUAD. There are required to precisely define the individual functions of lncRNAs in LUAD development in future.

## CONFLICT OF INTEREST

The authors declare that they have no competing interests.

## AUTHOR’S CONTRIBUTIONS

CL and HF conceived of the study. HLP, XQP, and XWM carried out the experiments, participated in the drafted the manuscript. XRD, LS, YHM, and LWT collected samples. HF and CL participated in the design of the study and helped to review the manuscript. HF, HLP, and HZJ performed the statistical analysis. All authors read and approved the final manuscript.

## Data Availability

The data sets used and/or analyzed during the present study are available from the corresponding author upon reasonable request.
